# Early vs. interval approach to laparoscopic cholecystectomy for acute cholecystitis: a retrospective observational study from Pakistan

**DOI:** 10.3389/fsurg.2024.1462885

**Published:** 2024-09-06

**Authors:** Sandesh Raja, Azzam Ali, Dileep Kumar, Adarsh Raja, Khursheed Ahmed Samo, Amjad Siraj Memon

**Affiliations:** ^1^ Department of Surgery, Dow Medical College, Dow University of Health Sciences, Karachi, Pakistan; ^2^ Department of Surgery, Dr. Ruth K. M. Pfau, Civil Hospital Karachi, Karachi, Pakistan; ^3^ Department of Surgery, Shaheed Mohtarma Benazir Bhutto Medical College Lyari, Karachi, Pakistan; ^4^ Department of Surgery, Jinnah Sindh Medical University, Karachi, Pakistan

**Keywords:** acute cholecystitis, laparoscopic cholecystectomy, early cholecystectomy, interval cholecystectomy, complications

## Abstract

**Background:**

Laparoscopic cholecystectomy (LC) is the preferred treatment for acute cholecystitis (AC). However, the optimal timing for LC in AC management remains uncertain, with early cholecystectomy (EC) and interval cholecystectomy (IC) being two common approaches influenced by various factors.

**Methods:**

This retrospective study, conducted at a tertiary care teaching hospital in Karachi, Pakistan, aimed to compare the outcomes of EC vs. IC for AC management. Patient data from January 2019 to September 2019 were analyzed with a focus on operative complications, duration of surgery, and postoperative hospital stay. The inclusion criteria were based on the Tokyo Guidelines, and patients underwent LC within 3 days of symptom onset in the EC group and after 6 weeks in the IC group.

**Results:**

Among 147 eligible patients, 100 underwent LC (50 in each group). No significant differences were observed in the sex distribution or mean age between the two groups. The EC group experienced fewer operative complications (12%) than the IC group (34%), with statistically significant differences observed. Nevertheless, no substantial variations in operative time or postoperative hospital stay were observed between the groups.

**Conclusion:**

Reduced complications in the EC group underscore its safety and efficacy. Nonetheless, further validation through multicenter studies is essential to substantiate these findings.

## Introduction

Acute cholecystitis (AC), a prevalent condition observed in emergency departments, often manifests as sudden abdominal pain. This is typically caused by a gallstone blocking the cystic duct, resulting in gallbladder (GB) inflammation (1). Once the diagnosis is established and the patient is deemed surgically fit for the procedure, laparoscopic cholecystectomy (LC) stands as the foremost treatment option for AC (2).

The best timing for the LC is still uncertain (3). AC is often treated using two different approaches that differ in timing involving LC. The first involves performing LC during the same hospitalization, usually within 3 days of symptom onset, referred to as early cholecystectomy (EC). The second option comprises conservative treatment initially, followed by interval cholecystectomy (IC) during a subsequent hospital admission, which is usually scheduled 3–9 weeks later (4–7). These approaches are influenced by institutional resources, the surgeon's skill level throughout the procedure, and the patient's overall health.

The concept of selecting IC over EC stems from surgeons’ concerns regarding the risks of transitioning to open surgery, common bile duct injury, and bleeding caused by acute inflammation. Acute inflammation presents complexities and obstacles in LC due to various factors, including swelling, fluid buildup, adhesions to adjacent structures, GB distension, delicate tissues, obscured and altered ductal and vascular anatomy, heightened blood flow, congestion, and the potential for infection dissemination (8). Furthermore, the management of emergency surgery is significantly complicated using anticoagulant medications. These medications increase the risk of bleeding and necessitate careful preoperative planning and adjustments to mitigate potential complications (9). Meanwhile, IC could increase the likelihood of additional complications related to gallstones during the interval period. Some patients might also choose to forego surgery after initial conservative treatment, being satisfied with medication results, which could subsequently elevate the risk of recurrence (10–13).

The primary benefit of EC is its provision of definitive treatment within the same hospital admission, thereby resulting in a shortened total hospital stay and cost reductions, whereas IC necessitates additional hospitalization (14). A recent meta-analysis conducted by Wu H et al. (3), which included 34 studies, concluded that there was no notable difference in operation duration and postoperative hospitalization, but there was a remarkable contrast in the total duration of hospitalization between the two strategies.

This study aimed to evaluate and compare outcomes to determine the preferred timing for AC in our context, with a specific focus on clinical endpoints. This was achieved by assessing intraoperative abdominal bleeding, bile duct injury, bile leak, conversion to open cholecystectomy, iatrogenic bowel injury, duration of the surgical procedure, and length of hospital stay in both groups.

## Methods

### Study design

This retrospective study included patients diagnosed with AC who underwent LC between January 2019 and September 2019 at a tertiary teaching hospital in Karachi, Pakistan, prior to the onset of the COVID-19 pandemic. Patient characteristics, hospitalization duration, and operative complications were obtained from the medical records. In reporting our study, we meticulously conformed to the principles and directives specified by the Strengthening the Reporting of Observational Studies in Epidemiology (STROBE) statement (15).

### Details of the intervention

Patients in the EC group underwent LC using the four-trocar technique within three days of AC onset, while those in the IC group underwent the procedure six weeks after AC onset. In the latter group, patients were initially administered medical treatment, and those who responded positively were subsequently discharged to undergo elective surgery six weeks later. LC was performed in both groups by the same general surgery team. Intravenous broad-spectrum antibiotics were administered to all the patients prior to surgery. Preoperative assessments of the patients were conducted considering factors such as age, sex, liver function tests (LFTs), abdominal ultrasound, and other baseline investigations.

### Selection criteria

A total of 147 patients were retrospectively identified as fulfilling the criteria for a confirmed diagnosis of AC, as outlined in the Tokyo Guidelines (16), which includes the presence of specific clinical symptoms and imaging findings. To confirm the diagnosis of AC according to the Tokyo Guidelines, one or more clinical features, such as acute right upper quadrant pain, tenderness, a positive Murphy's sign, fever (>37.5°C), or leukocytosis (>10,000 /mm^3^), along with at least one imaging finding, such as the presence of gallstones, a thickened gallbladder wall (>3 mm), pericholecystic fluid, or a positive sonographic Murphy's sign, are required. Patients were excluded if they had pancreatitis, cholangitis, peritonitis, or concurrent cholecystitis with choledocholithiasis, even if they met the criteria for AC. These conditions, related to pancreatic-biliary pathology, could have affected the study outcomes. Pregnant women and individuals who opted out of IC after successful medical therapy were excluded from this study. Consequently, 47 patients were not included in this study.

### Outcome assessment

The main objective of this study was to assess operative complications in both groups. These involved assessing the rates of intraoperative abdominal bleeding, bile duct injury, bile leak, conversion to open cholecystectomy, iatrogenic bowel injury, surgical site infection, and 30-day postoperative mortality. Additionally, the duration of the surgical procedure and postoperative hospital stay were compared as secondary outcomes between the two groups.

### Statistical analysis

Data were collected and processed using SPSS version 22 (IBM Corp.). For continuous variables, means and standard deviations were documented, whereas frequencies were determined for categorical variables using descriptive statistics Univariate analysis of the two-group categorical values was performed using the chi-square test. The Mann–Whitney *U* test was employed for univariate analysis of continuous variables. Statistical significance was set at *P* < 0.05.

### Ethical considerations and informed consent

The study adhered to ethical guidelines and obtained approval from the Institutional Review Board in accordance with the principles outlined in the Declaration of Helsinki. Owing to the retrospective nature of the study, the need for informed consent was waived as it involved the review of historical data.

## Results

### Patient characteristics

**Figure 1:**
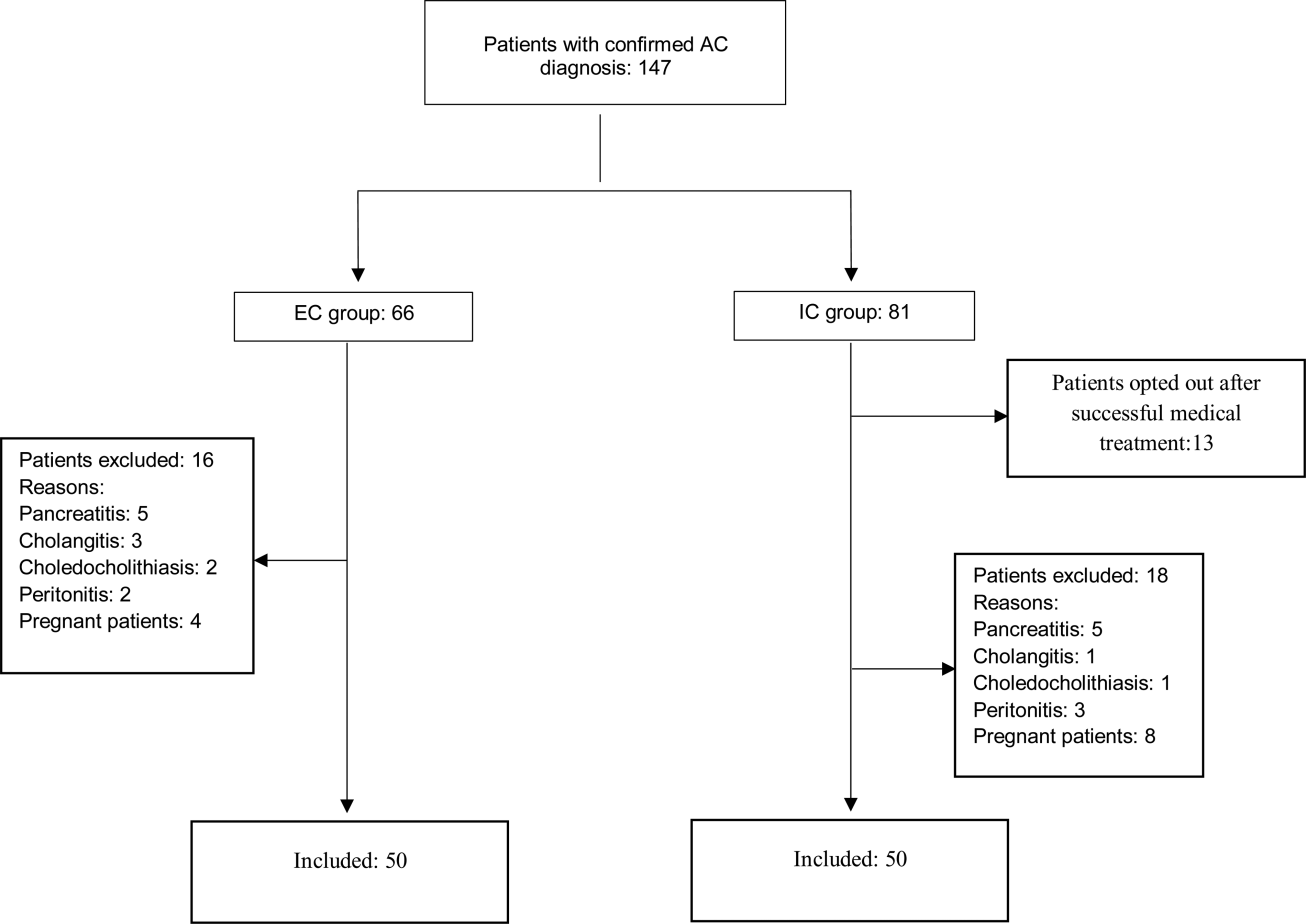
Flow diagram.

The study was conducted prior to the onset of the COVID-19 pandemic. We identified 147 diagnosed cases of AC between January 2019 and September 2019. Among these, 66 patients underwent EC, and 68 patients underwent IC. This resulted in a final cohort of 100 patients, with 50 in each group—a balanced distribution that occurred by coincidence (Figure 1). Upon comparing patient characteristics, no significant disparities were observed in the distribution of sex between the two groups (*p* = 0.216), with 44% male and 56% female in the EC group, and 32% male and 68% female in the IC group. Similarly, there was no statistically significant difference in mean age between the two groups (*p* = 0.184), with an average age of 48.9 years in the EC group and 51.3 years in the IC group (Table 1).

**Table 1:** Patient characteristics and operative parameters (findings/timing).

Characteristics	EC (<3 days) (*n* = 50)	IC (>6 weeks) (*n* = 50)	*p* value
Sex (M/F)	22/28	16/34	0.216
Age (years) (Mean ± SD)	48.9 ± 7.81	51.3 ± 8.42	0.184
Total operative findings (*N*) (%)	18 (36%)	50 (100%)	<0.001
Adhesions	13 (26%)	2 (4%)	0.002
Contracted GB	0	20 (40%)	<0.001
Densely Adherent GB	1 (2%)	18 (36%)	<0.001
Distended GB with pus or mucus	4 (8%)	10 (20%)	0.083
Duration of LC (minutes) (Mean ± SD)	86 ± 17.6	93 ± 20.1	0.091

EC, early cholecystectomy; IC, interval cholecystectomy; LC, laparoscopic cholecystectomy; GB, gallbladder; SD, standard deviation.

### Operative findings

During LC, various findings were observed among patients in both groups. Notable findings included the presence of adhesions, densely adherent GB, contracted GB, and distended GB with pus or mucus.

Adhesions were found in 26% of patients in the EC group compared to only 4% in the IC group, representing a significant disparity (*p* = 0.002). Although contracted GB was not observed in the EC group, it was found in 40% of the cases in the IC group, indicating a significant difference (*p* < 0.001). Densely adherent GB was observed in 2% of the patients in the EC group vs. 36% in the IC group, indicating a noteworthy difference (*p* < 0.001). As for distended GB with pus or mucus, 8% of cases in the EC group and 20% in the IC group displayed this feature. Nonetheless, there was no statistically significant contrast between the groups (*p* = 0.083) (Table 1).

### Operative time

Operative time was compared between the groups. The mean duration was 86 min for EC and 93 min for IC. Although the latter showed a trend towards longer lengths, the difference was not statistically significant (*p* = 0.091) (Table 1).

### Outcome

#### Operative complications

The EC group experienced a total of six complications (12%), whereas the IC group experienced 17 complications (34%). This difference in overall complication rates was statistically significant (*p* = 0.009). Specifically, the EC group had two cases of conversion to open cholecystectomy (4%), one case of bleeding (2%), one case of common bile duct (CBD) injury (2%), one case of bile leak (2%), and one case of iatrogenic bowel injury (2%). In comparison, the IC group had four cases of conversion to open cholecystectomy (8%), six cases of hemorrhage (12%), one case of CBD injury (2%), four cases of bile leak (8%), and two cases of iatrogenic bowel injury (4%).

However, it is important to note that while the overall complication rates were significantly higher in the IC group, the individual complication rates did not show significant differences between the two groups. The *p*-values for conversion to open cholecystectomy (0.400), hemorrhage (0.050), CBD injury (1.00), bile leak (0.169), and iatrogenic bowel injury (0.558) were statistically insignificant. Additionally, there were no reported cases of surgical site infection or 30-day postoperative mortality in either group (Table 2).

**Table 2:** Operative complications and post-operative stay.

	EC (<3 days) (*n* = 50)	IC (>6 weeks) (*n* = 50)	*p* value
Total complications (*N*)	6	17	*0*.*009*
Conversion to open LC	2	4	0.400
Bleeding	1	6	0.05
CBD injury	1	1	1.00
Bile leak	1	4	0.169
Iatrogenic bowel injury	1	2	0.558
Post-operative stay (day) (Mean ± SD)	(3.89 ± 0.74)	(4.09 ± 0.82)	0.214

EC, early cholecystectomy; IC, interval cholecystectomy; LC, laparoscopic cholecystectomy; CBD, common bile duct.

### Cross tabulation: complications and operative findings

Cross-tabulation of complications against operative findings highlights distinct patterns between the IC and EC groups. Specifically, 8% of cases in the IC group required conversion to open surgery, with contracted GB being associated with these cases, as opposed to 4% in the EC group, which was linked to a distended GB with pus or mucus. Furthermore, in the IC group, 12% of the bleeding instances were related to contracted GB, whereas only 2% in the EC group were associated with adhesions. Similarly, 8% of bile leak events in the IC group were related to a densely adherent GB, compared to 2% in the EC group, who had a distended GB with pus or mucus. In addition, 2% of cases of CBD injury in the IC group had contracted GB, whereas 2% in the EC group had adhesions. Additionally, 4% of iatrogenic bowel injury cases in the IC group had contracted GB (2%) and distended GB with pus or mucus (2%), whereas 2% in the EC had distended GB with pus or mucus (Table 3).

**Table 3:** Cross tabulation: complications and operative findings.

Complications	Operative findings							
	Adhesion		Densely adherent GB		Contracted GB		Distended GB with pus or mucus	
	EC	IC	EC	IC	EC	IC	EC	IC
CBD injury	1	–	–	–	–	1	–	–
Bleeding	1	–	–	–	–	6	–	–
Iatrogenic Bowel Injury	–	–	–	–	–	1	1	1
Bile leak	–	–	–	4	–	–	1	–
Conversion to open	–	–	–	–	–	4	2	–

EC, early cholecystectomy; IC, interval cholecystectomy; CBD, common bile duct; GB, gallbladder.

### Postoperative hospital stay

The average duration of postoperative stay was 3.89 days in the EC group and 4.09 days in the IC group. Nevertheless, there was no statistically significant contrast between the two groups regarding postoperative hospital stay (*p* = 0.214) (Table 2).

## Discussion

In this retrospective investigation, we found that EC for AC significantly reduced overall complications compared to IC (*p* = 0.009). Moreover, there were no significant differences between the two groups in terms of the operative duration and postoperative hospital stay.

Following the success of the first LC in the late 1980s, minimally invasive surgery has become more common for the treatment of biliary tract diseases and the primary surgical method for cholecystectomy (17). In the early days of laparoscopic surgery, AC was considered a relative contraindication to LC (18), with the best timing for the procedure being 6 to 8 weeks following the acute phase to allow resolution of GB inflammation (19). As expertise in laparoscopic procedures increased, iatrogenic bile duct injuries and conversion rates declined. However, the optimal timing for LC in patients with AC remains controversial. Several clinical studies, ranging from prospective to retrospective investigations and meta-analyses, have demonstrated that EC is safe and results in a shorter duration of hospitalization and lower costs. Additionally, the rates of complications associated with EC are equivalent to or even better than those reported for IC (3, 6, 7, 20–25). Hence, this investigation aimed to assess the safety of EC and IC for AC in our specific setting.

Early research indicated a correlation, with rates ranging from 6% to 35%, in cases requiring conversion to open surgery when EC was performed for AC (18). Recent data indicate that the rates of conversion to open surgery are either similar or slightly elevated among patients who undergo IC. In a randomized controlled trial conducted by Gutt et al. (26), 33 instances (11.9%) of conversion to open laparotomy were observed in the IC group compared to 30 cases (9.9%) in the EC group (*p* = 0.44). This aligns with our own findings, where we noted four conversions (8%) in the IC group and two (4%) in the EC group, with a *p*-value of 0.400. Budişcă OA et al. (7) recently also showed that the IC group had greater rates of conversion to open surgery than the EC group. Only a few studies have reported the incidence of intraoperative bleeding. These studies consistently indicated that both groups exhibited a comparable number of intraoperative bleeding cases, showing no significant difference between the two groups. Based on the findings of Kohga A et al. (23), Gutt CN et al. (26), and Budişcă OA et al. (7) reported one instance of bleeding in both the EC and IC groups, and Ozkardeş et al. (22) documented one case of bleeding in the EC group and none in the IC group. Our study revealed more bleeding cases in the IC group (six cases) compared to 1 in the EC group (one case). Contracted GB in the most IC group increased bleeding risk, resulting in open surgery in 3 patients. However, this disparity was not statistically significant (*p*-value = 0.05).

In our investigation, we discovered no substantial distinction in the occurrence of common bile duct injury and bile leak between the intervention groups, with *p*-values of 1.00 and 0.169, respectively. The study conducted by. (21) Yielded congruent results, indicating the absence of statistically significant disparities in the incidence rates of both common bile duct injury (*p* = 0.43) and bile leakage (*p* = 0.78) between the EC and IC intervention groups. The meta-analyses conducted by Wu XD et al. (27) and Lyu Y et al. (28) largely corresponded with our study findings, particularly concerning common bile duct injury and bile leakage, and revealed no discernible discrepancy between the EC and IC groups. Iatrogenic bowel injury is a complication of laparoscopic surgery. Our investigation documented a collective total of three instances of iatrogenic bowel injury, with one occurrence in the EC group and two in the IC group. In the EC group, bowel injury correlated with a distended GB containing pus, necessitating open surgery in one patient. Conversely, within the IC group, bowel injury was linked to a contracted GB, resulting in open surgery in one patient, whereas another patient exhibited bowel injury associated with a distended GB containing pus.

EC for AC is associated with a shorter hospitalization duration. Nonetheless, existing data suggest that there is no statistically significant difference between EC and IC in terms of both postoperative hospital stay and operation time (3, 7, 29). This study revealed no notable differences between the groups in terms of postoperative hospital stay (*p* = 0.214) and operation time (*p* = 0.091).

Our study's findings should be interpreted considering several limitations. The retrospective design and reliance on data from a single institution may constrain the generalizability of our results. The absence of long-term follow-up data could also affect the strength of our conclusions. Additionally, the small sample size, lack of cost analysis, and the limited timeframe of the study period from January 2019 to September 2019 are notable constraints. Specifically, the retrospective nature of this study, coupled with the limitations inherent in a resource-scarce setting, restricted our ability to account for all relevant variables, such as patients’ concomitant chronic diseases (e.g., diabetes, hypertension, coronary artery disease). These factors could have influenced surgical outcomes and overall morbidity and mortality. Furthermore, due to the retrospective design of the study, there is no available data on criteria such as adhesions, contracted gallbladder, densely adherent gallbladder, and distended gallbladder with pus or mucus. Future research should aim to address these limitations through multicenter studies with extended follow-up periods and comprehensive cost analyses to better assess the efficacy of laparoscopic surgery in emergency settings across diverse clinical environments.

## Conclusion

Our retrospective investigation of EC and IC provides valuable insights into the optimal timing of LC in treating AC. We observed notable distinctions in the overall operative complications between the two groups, with the EC group demonstrating decreased rates. Nonetheless, no significant variances were detected in the operative duration and postoperative hospitalization. These results emphasize the safety and effectiveness of EC for addressing AC.

## Data Availability

The original contributions presented in the study are included in the article/Supplementary Material, further inquiries can be directed to the corresponding authors.
